# Safety and Efficacy of First-Line Treatments for Chemotherapy-Naive Metastatic Castration-Resistant Prostate Cancer: A Systematic Review and Indirect Comparison

**DOI:** 10.1155/2017/3941217

**Published:** 2017-12-07

**Authors:** Haofeng Zheng, Jialiang Chen, Wenhan Qiu, Sijie Lin, Yanxiong Chen, Guancan Liang, Youqiang Fang

**Affiliations:** Department of Urology, The Third Affiliated Hospital of Sun Yat-Sen University, Guangzhou 510630, China

## Abstract

Recently, several drugs have been introduced for the first-line treatment of chemotherapy-naive metastatic castration-resistant prostate cancer (mCRPC), but few studies have compared treatment outcomes directly. This indirect comparison among 10 clinical trials (*n* = 4870 patients) retrieved from PubMed, Web of Science, Cochrane Collaboration, and ClinicalTrails.gov was performed to assess the safety and efficacy of docetaxel, cabazitaxel, abiraterone, enzalutamide, and sipuleucel-T for the initial treatment of mCRPC. No significant differences in primary outcome (overall survival) were found among initial treatments. However, docetaxel had the highest probability (37.53%) of being the most effective, but at the cost of more adverse events, while enzalutamide was associated with the best secondary outcomes (prostate-specific antigen response, progression-free survival, quality of life, and adverse event profile). Thus, docetaxel is recommended as the first agent used for the chemotherapy of mCRPC, while enzalutamide is recommended as the first nonchemotherapy treatment. Additional clinical trials are needed to confirm these findings and establish the optimal order for multidrug treatment of mCRPC.

## 1. Introduction

Prostate cancer (PCa) is one of the most commonly diagnosed solid organ malignancies in the United States (US) and the third leading cause of cancer death among American men [1]. It is projected that more than 161,000 new PCa diagnoses and over 26,000 deaths will occur in the United States during 2017 [2]. Almost all cases progress to metastatic castration-resistant prostate cancer (mCRPC), for which the median overall survival time is always within two years [3].

Androgen-deprivation therapy is the first treatment strategy used for advanced PCa, but this treatment only slows progression [4]. As progression to mCRPC is the main cause of death from PCa, effective therapies for mCRPC are of vital importance for overall survival [5]. According to the 2017 guidelines of the European Association of Urology (EAU) [6], several drugs, including docetaxel, abiraterone, enzalutamide, and sipuleucel-T, are recommended as first-line treatments for mCRPC, while cabazitaxel is recommended for second-line treatment (if not superior to docetaxel as first-line treatment).

Although many clinical trials have investigated first-line treatments for mCRPC, few have compared these drugs directly. Therefore, it is difficult for urologists to decide which drug to use first. Hence, this indirect comparison among clinical trials was performed to assist clinicians as well as PCa researchers planning treatment trials.

## 2. Materials and Methods

The protocol for this indirect comparison was registered at PROSPERO (42017069009). Details of the protocol can be accessed at https://www.crd.york.ac.uk/prospero/display_record.asp?ID=CRD42017069009. The protocol adhered strictly to the preferred reporting items for systematic reviews and meta-analyses (PRISMA) statement (Supplemental Table 1) [7].

### 2.1. Study Selection and Data Collection

Only randomized controlled trials (RCTs) and phase-3 clinical trials (CTs) comparing any of the following six drugs, docetaxel, cabazitaxel, mitoxantrone, abiraterone, enzalutamide, and sipuleucel-T, as initial treatment for mCRPC in adult males (18 years or older) were included. Clinical trials that focused on treatment of patients after failed docetaxel therapy or chemotherapy were excluded.

PubMed, Web of Science, Cochrane Collaboration, and ClinicalTrials.gov were searched to identify relevant studies up to June 29, 2017. Reference lists were also searched for related articles. Titles and abstracts were first used to screen articles, and then full-text reviews were used for the final decision. Quality and bias of publication were assessed using the Cochrane Risk of Bias tool. Two reviewers (H. F. Z. and J. L. C.) completed this process, and all disagreements were settled by a senior author (Y. Q. F.).

Data were first gathered from the results of ClinicalTrials.gov and then updated by the relevant articles. The complete or most recent research report was used when several studies involved the same population.

### 2.2. Outcome Measures

Primary outcome was overall survival (OS) and secondary outcomes were prostate-specific antigen (PSA) response and adverse events (AEs). In addition, progression-free survival (PFS) or radiographic PFS (rPFS), time to tumor progression (TTP), PSA-progression free survival (PSA-PFS), and health-related quality of life (HRQL) were used in subgroup analyses. Detailed definitions of these outcomes can be found in our online protocol.

### 2.3. Comparability of Dosages

Considering the goals of this research, only trials that used the dosages recommended by the EAU were included. A lower dose of cabazitaxel (20 mg/m^2^) was included because efficacy was similar to a higher dose (25 mg/m^2^) but with lower toxicity in one study [8].

### 2.4. Statistical Analysis

Hazard ratio (HR) was used to assess most outcomes (OS, PFS, rPFS, TTP, PSA-PFS, and HRQL) because it provides time-to-event information with adjustment for confounders. If the HR could not be obtained from the research directly, it was estimated using the method described by Tierney et al. [9]. For binary outcomes, including PSA response rate and adverse event rate, risk ratio (RR) was used, as the CTs included were all prospective studies. All outcomes measures include the 95% confidence interval (95% CI). The top 10 most frequent AEs were also analyzed to evaluate drug safety.

Indirect comparisons were conducted using WinBUGS version 1.4.3 (MRC Biostatistics Unit, Cambridge, UK). Normal prior distributions, noninformative uniform, and 3 different sets of starting values were used to fit the model. In order to obtain the posterior distributions of model parameters, 150,000 iterations (50,000 per chain) were yielded. For each chain, 20,000 burn-ins and a thinning interval of 10 were used. Efficacies of anti-mCRPC drugs were ranked by calculating the HR or RR compared to placebo, docetaxel, or mitoxantrone. Other outcomes were ranked by the same method.

Review Manager 5.3 (Cochrane Collaboration, Oxford, UK) was used to present the results of indirect comparisons and to conduct a traditional pairwise meta-analysis. A *p* < 0.05 was considered statistically significant for all tests.

## 3. Results

### 3.1. Literature Search

**Figure 1:**
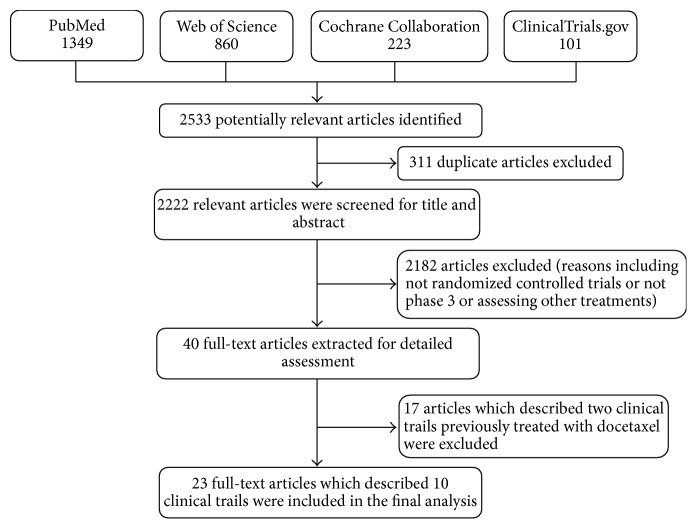
Flow diagram of study identification, inclusion, and exclusion.

A total of 2533 potentially relevant articles were identified after the initial database search. After excluding 2510 articles not meeting inclusion criteria, 23 full-text articles which described 10 CTs, including 3 for docetaxel [10–12], 3 for sipuleucel-T [13–15], 1 for abiraterone [16, 17], 1 for enzalutamide [18, 19], 1 for mitoxantrone [20], and 1 for cabazitaxel [8], were included in the final analysis (Figure 1). Agreement between the two researchers was 100% for quality assessment of included CTs.

### 3.2. Characteristics of Eligible Studies

**Figure 2:**
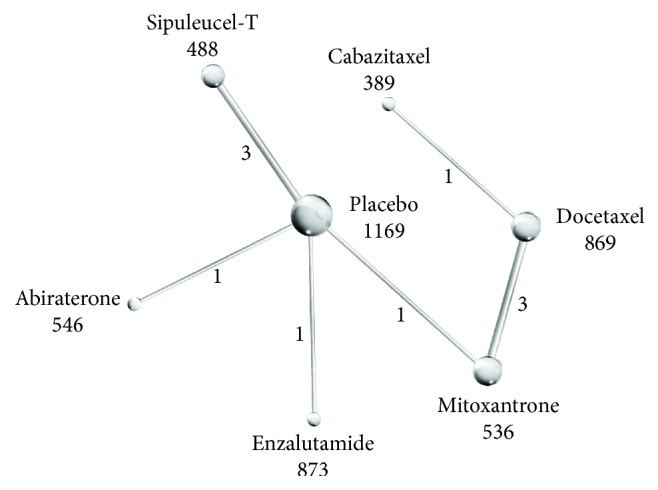
Network of indirect comparisons. The size of the nodes indicates the number of patients (listed under the nodes) and line width the number of trials comparing each pair of treatments (listed under the lines).

Table 1 summarizes the details of CTs included in this study, and interactions of interventional treatments included in this indirect comparison are shown in Figure 2. All CTs were multicenter except that by Shen et al. comparing docetaxel to mitoxantrone [10]. No significant differences in baseline patient characteristics were found between treatment and control groups in the initial study. All CTs were deemed to be of high quality, although the protocols of two could not be found, which may introduce attrition bias (Supplemental Figure 1).

### 3.3. Primary Outcome

**Figure 3:**
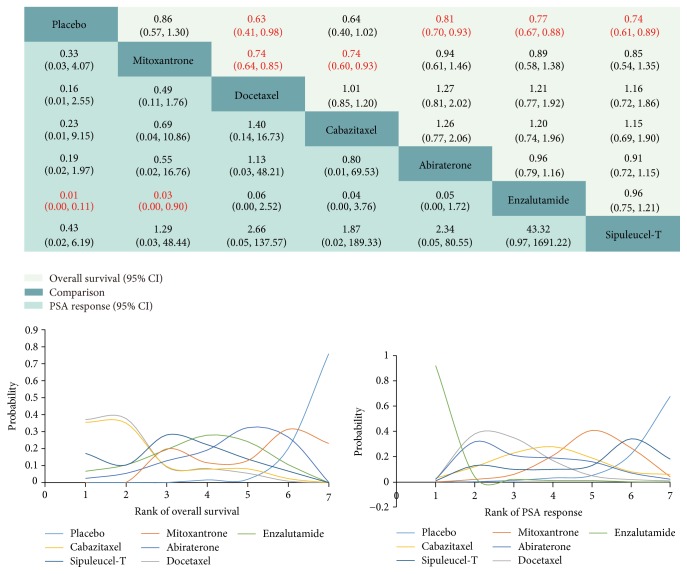
Overall survival and PSA response for the included comparisons. The hazard ratio (HR) is used to express differences in overall survival (column treatment versus row treatment), with HR < 1 favoring column treatment. For PSA response, risk ratio (RR) is used (row treatment versus column treatment) with RR < 1 favoring row treatment. Text in red indicates a significant difference (*p* < 0.05). First rank indicates highest probability of greatest efficacy as determined by overall survival (OS) or PSA response. PSA: prostate-specific antigen.

Results of the OS comparisons, including HRs and ranks, are presented in Figure 3. All treatments were found to improve OS compared to placebo except mitoxantrone (HR 0.86, 95% CI 0.57 to 1.30) and cabazitaxel (HR 0.64, 95% CI 0.40 to 1.02). When compared to docetaxel, however, no significant differences were found among cabazitaxel (HR 1.01, 95% CI 0.85 to 1.20), abiraterone (HR 1.27, 95% CI 0.81 to 2.02), enzalutamide (HR 1.21, 95% CI 0.77 to 1.92), and sipuleucel-T (HR 1.16, 95% CI 0.72 to 1.86). Docetaxel exhibited the highest probability (37.53%) of being the most effective drug for OS among those compared.

### 3.4. Secondary Outcomes

#### 3.4.1. Prostate-Specific Antigen Response

Enzalutamide demonstrated a higher PSA response rate compared to both mitoxantrone (RR 0.03, 95% CI 0.00 to 0.90) and placebo (RR 0.01, 95% CI 0.00 to 0.11), while no obvious differences were found among other comparisons (Figure 3). Enzalutamide showed the highest probability (92%) of ranking first among included drugs regarding PSA response.

#### 3.4.2. Adverse Events

**Figure 4:**
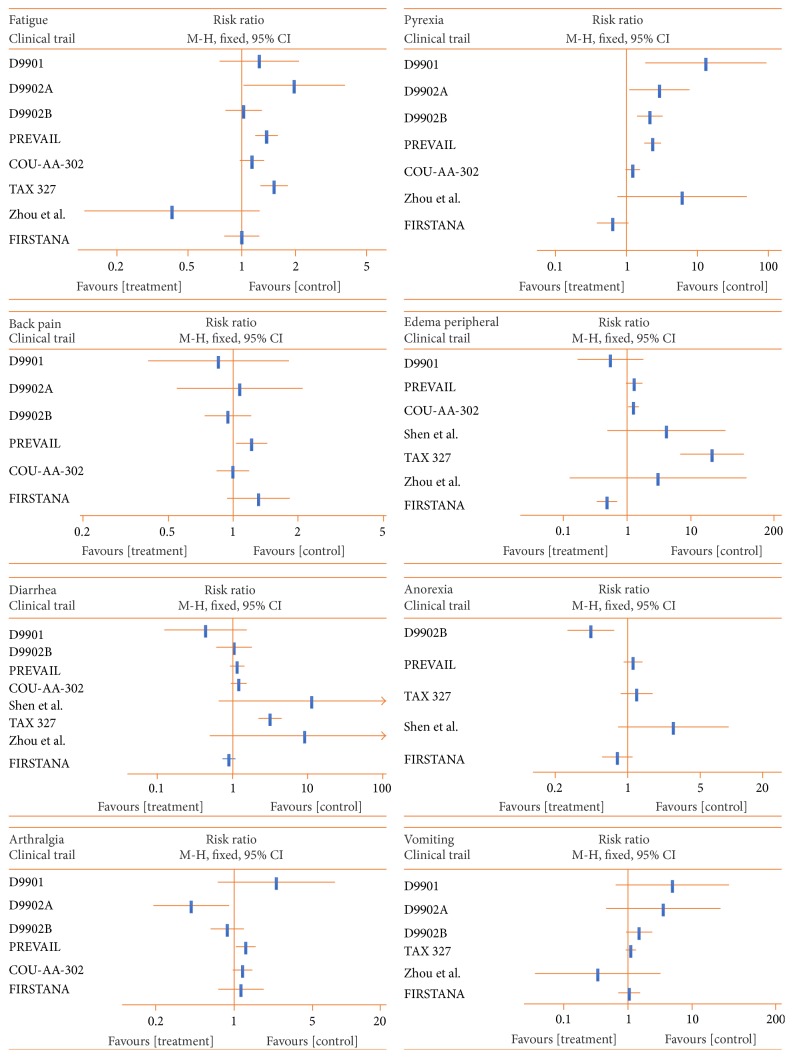
Top 10 most frequent adverse events (EAs) among all clinical trials. Fatigue ranks first, back pain ranks second, and vomiting ranks last. Risk ratio (RR) is used to express the difference in AEs, with RR < 1 favoring the treatment.

The top 10 most frequent AEs among all CTs (Figure 4) were as follows: fatigue, back pain, diarrhea, constipation, arthralgia, pyrexia, edema peripheral, nausea, anorexia, and vomiting. Serious AEs are listed in Supplemental Figure 2.

### 3.5. Subgroup Analysis

#### 3.5.1. Chemotherapy

**Figure 5:**
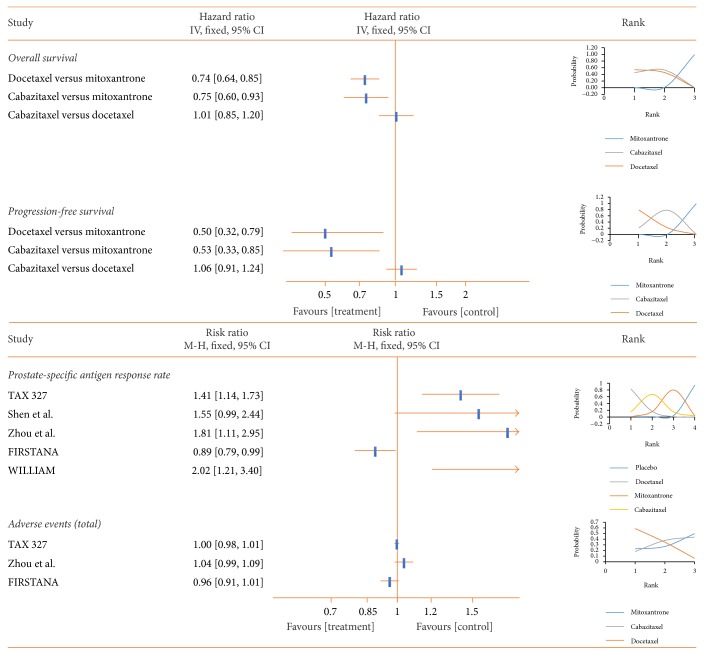
Pooled analysis of the chemotherapy subgroup (mitoxantrone, docetaxel, and cabazitaxel). Hazard ratio (HR) is used to indicate differences in overall survival (OS) and progression-free survival (PFS), while risk ratio (RR) is used for PSA response and adverse events (AEs). An HR or RR < 1 favors that treatment group. First rank indicates superior outcome (excluding AEs). PSA: prostate-specific antigen.

Docetaxel was associated with the highest OS (HR 0.74, 95% CI 0.64 to 0.85), PFS (HR 0.50, 95% CI 0.32 to 0.79), and PSA response rate (RR 0.49, 95% CI 0.11 to 1.76) among the three chemotherapy drugs when compared to mitoxantrone. However, docetaxel was also associated with the most AEs among chemotherapy drugs (RR 0.64, 95% CI 0.15 to 2.71) when compared to mitoxantrone (Figure 5).

#### 3.5.2. Nonchemotherapy

**Figure 6:**
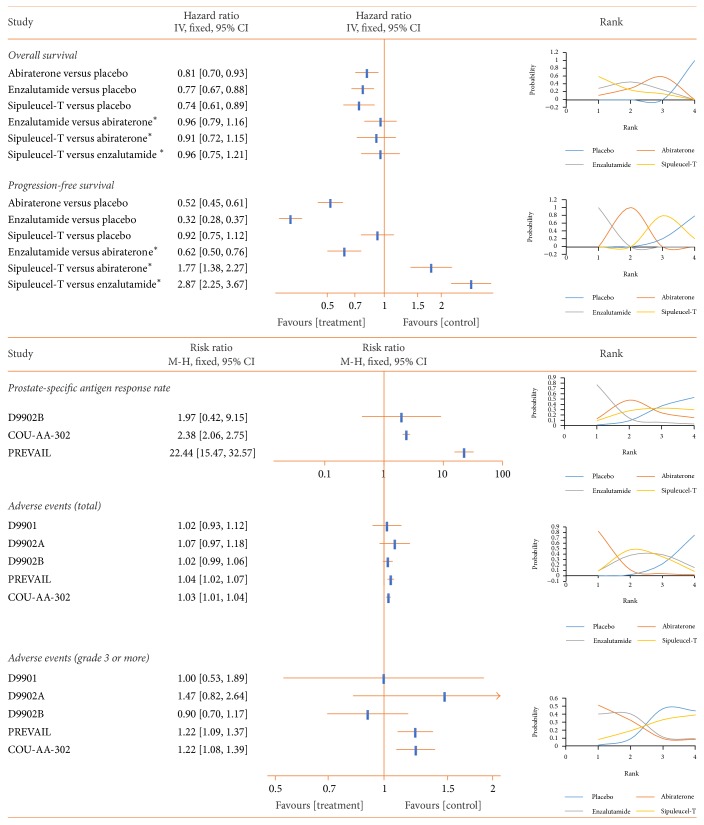
Pooled analysis of the nonchemotherapy subgroup (abiraterone, enzalutamide, and sipuleucel-T). Hazard ratio is used to indicate differences in overall survival (OS) and progression-free survival, while risk ratio (RR) is used for PSA response and adverse events. An HR or RR < 1 favors that treatment group. First rank means superior outcome (excluding AEs). PSA: prostate-specific antigen. ^*∗*^Indirect comparison.

Sipuleucel-T showed the highest probability (59.4%) of being the most efficacious for OS improvement among the three nonchemotherapy drugs (HR 0.74, 95% CI 0.61 to 0.89) compared to placebo, while enzalutamide yielded the best PFS (HR 0.32, 95% CI 0.28 to 0.37), the best PSA response (RR 0.01, 95% CI 0.00 to 3.32), and the fewest AEs (RR 0.38, 95% CI 0.02 to 6.87) when compared to placebo (Figure 6).

## 4. Discussion

This indirect comparison of first-line treatments for chemotherapy-naive mCRPC across 10 CTs (4870 patients) suggests that docetaxel has the greatest potential efficacy as indicated by OS, while cabazitaxel shows no obvious difference in efficacy but causes fewer AEs than docetaxel. Of nonchemotherapy drugs included for comparison, enzalutamide shows the highest probability for superior OS and PFS as well as fewest AEs. Therefore, docetaxel is recommended as the first-line chemotherapy and enzalutamide as the first-line nonchemotherapy treatment for advance prostate cancer.

Based on this indirect comparison of multiple chemotherapy and nonchemotherapy drugs, chemotherapy appears to be the best choice for initial treatment of mCRPC, although there was no significant difference among first-line treatments. Docetaxel and cabazitaxel both bind to and stabilize tubulin, inhibiting microtubule depolymerization and resulting in tumor cell cycle arrest and apoptosis [21]. The apparent superior efficacy of chemotherapy over nonchemotherapy may result from nonspecific targeting of multiple cell types. At the same time, however, such nonspecificity could lead to increased AEs, and indeed AEs were more frequent in the chemotherapy than nonchemotherapy group.

Docetaxel 75 mg/m^2^ every three weeks combined with prednisone 5 mg twice daily is one first-line treatment recommended by the EAU, but serious side effects are a substantial issue with this regimen [11]. A recent phase-3 trial reported that 50 mg/m^2^ docetaxel administered every 2 weeks could improve OS and time to treatment failure compared to 75 mg/m^2^ every three weeks [22]. Similarly, a phase-3 noninferiority study of mCRPC patients previously receiving docetaxel found that 20 mg/m^2^ cabazitaxel had efficacy equal to 25 mg/m^2^ for OS with lower toxicity [23]. Therefore, lower, less toxic doses may be possible, but additional CTs are needed to address the optimal dose regimen and rank efficacy of chemotherapy drugs for mCRPC.

Prostate-specific antigen response is commonly used in CTs as an efficacy measure for mCRPC response, but the clinical significance of the PSA response is unclear [24]. Higher PSA response rate (>50% decline in PSA from pretreatment baseline) was associated with longer survival time in one study [25], but the rank order of PSA responses was not consistent with that for OS among the drugs evaluated in the current study. This inconsistency was especially large for sipuleucel-T, possibly due to distinct drug mechanisms as sipuleucel-T is an immunotherapeutic drug rather than a direct cancer cell toxin. The PSA response rate of sipuleucel-T was quite low among the three relevant CTs, but sipuleucel-T was the third most efficacious drug for enhancing OS. Thus, the immune response is likely a more relevant index of antitumor activity than PSA response for sipuleucel-T treatment.

While prolonging life is the primary aim of cancer therapy, treatment decisions must also account for quality of life. Most mCRPC patients have no noticeable tumor-related symptoms initially and might not be suitable for chemotherapy. Therefore, new drugs or nonchemotherapy drugs with better side effects profiles are recommended. In fact, nonchemotherapy drugs such as abiraterone, enzalutamide, and sipuleucel-T are recommended by the EAU as first-line treatments for mCRPC [6], and CTs have shown notable benefits of these treatments for OS, PFS, and PSA response in addition to AEs compared to placebo or prednisone. Both abiraterone and enzalutamide target androgen-receptor (AR) signaling pathways [17, 26], while sipuleucel-T is a kind of cellular vaccine that targets PCa cells expressing prostate acid phosphatase (PAP) [13]. Although no obvious differences were found in overall efficacy among these nonchemotherapy drugs, sipuleucel-T appears to be the better first-line treatment for prolonging OS, while enzalutamide appears more favorable for rPFS/PFS, PSA response, AEs, PSA-PFS, and HRQL. Differences in clinical responses between sipuleucel-T and AR-related drugs may also stem from distinct cellular mechanisms (AR signaling inhibitor versus immune modulator). Most AEs associated with immune therapies, including chills, fever, fatigue, nausea, and headache, occur within one day after infusion, and are resolved within one to two days.

The results of our indirect comparison of the AR-related drugs abiraterone and enzalutamide are consistent with previous indirect comparisons [27, 28] (Supplemental Figure 3) including both chemotherapy-naive and postchemotherapy patients. However, differences between these drugs may stem from the comparator used. The enzalutamide CT used a true placebo group while controls in the abiraterone CT took prednisone 5 mg twice daily. A meta-analysis by Charity and associates suggested that prednisone cannot prolong the lives of mCRCP patients but can enhance quality of life [29]. Prednisone may thus obscure outcome differences between abiraterone and control groups. Clearly, additional studies comparing abiraterone and enzalutamide are needed.

Results of this indirect comparison of nonchemotherapy drugs are consistent with the latest clinical trials presented at the 2017 American Society of Clinical Oncology (ASCO). A randomized phase-2 cross-over study by Kim N. Chi comparing abiraterone plus placebo versus enzalutamide found no notable difference in time to PSA progression or time to tumor progression. Further, the efficacy of abiraterone was confirmed by several important clinical trials, including LATITUDE and STAMPEDE. With the assistance of androgen-deprivation therapy, abiraterone was found to improve the OS of locally advanced as well as metastatic PCa patients [30, 31]. Thus, abiraterone may improve OS not only in locally advanced patients but also in metastatic castration-resistant prostate cancer patients (detailed information can be found in the prostate cancer section of ASCO at https://www.urotoday.com/conference-highlights/asco-2017/asco-2017-prostate-cancer.html).

Therapy for mCRPC is a systematic process, and all treatments will ultimately fail [32]. Therefore, rational use of multiple drugs is of vital importance. The optimal order of drugs has also been investigated [33]. Shameem and colleagues reported that postchemotherapy may lessen the efficacy of abiraterone in patients with mCRPC. Thus additional CTs should focus on these first-line treatments, especially enzalutamide, including comparison with other first-line treatments and investigation of sequential treatment order.

The main limitation of this study is that all comparisons were across CTs, so the evidence level can be regarded as equivalent to a retrospective study. Second, several methodological features of these CTs varied (Table 1), especially follow-up duration, which may introduce bias. Finally, the number of CTs compared was limited, although these studies collectively encompassed a fairly large patient cohort. Nonetheless, this is the first study to compare the safety and efficacy of all EAU-recommended first-line treatments for chemotherapy-naive mCRPC patients and provide preliminary evidence for the optimal starting treatment strategy.

## 5. Conclusions

In conclusion, docetaxel is likely to be the most effective drug for the chemotherapy of mCRPC and so is recommend as first-line treatment, while enzalutamide is the recommended first-line nonchemotherapeutic drug. Further clinical trials are required to confirm these results and to establish the optimal order of drug administration for mCRPC.

## Figures and Tables

**Table 1:** Characteristics of clinical trials included in this study.

Clinical trail	Design	Sources of patients	Follow-up (median, month)	Intervention	Control	Number of patients^*∗*^	Age (median, years)^*∗*^	Gleason score (median)	PSA level (median, ng/ml)	ECOG PS (range)
D9902B NCT00065442	Phase 3	75 centers in the United States and Canada	34.1	Sipuleucel-T (3 complete doses/2 weeks)	APC placebo (3 complete doses/2 weeks)	341:171	72:70	NR	NR	0 to 1

D9901 NCT00005947	Phase 3	19 centers in the United States	36	Sipuleucel-T (3 complete doses/2 weeks)	APC placebo (3 complete doses/2 weeks)	82:45	73:71	7:7	46.0:47.9	0 to 1

D9902A NCT01133704	Phase 3	24 clinical trial sites	36	Sipuleucel-T (3 complete doses/2 weeks)	APC placebo (3 complete doses/2 weeks)	65:33	70:71	NR	61.3:44.0	0 to 1

PREVAIL NCT01212991	Phase 3	207 sites in 22 countries	31	Enzalutamide (160 mg/d)	Placebo	872:845	72:71	NR	54.1:44.2	0 to 1

COU-AA-302 NCT00887198	Phase 3	12 countries	49.2	Abiraterone (1000 mg/d) plus prednisone (10 mg/d)	Prednisone (10 mg/d)	546:542	71:70	NR	42:37.7	0 to 1

TAX 327 Tannock et al.	Phase 3	24 countries	20.8	Docetaxel (75 mg/m^2^)/3 weeks plus prednisone (10 mg/d)	Mitoxantrone (12 mg/m^2^)/3 weeks plus prednisone (10 mg/d)	335:337	68:68	NR	114:123	NR

Shen et al.	RCT	Single center	16.8	Docetaxel (75 mg/m^2^)/3 weeks plus prednisone (10 mg/d)	Mitoxantrone (12 mg/m^2^)/3 weeks plus prednisone (10 mg/d)	30:31	64:66	NR	63.45:100.86	NR

Zhou et al. NCT00436839,	RCT	15 centers in China	60	Docetaxel (75 mg/m^2^)/3 weeks plus prednisone (10 mg/d)	Mitoxantrone (12 mg/m^2^)/3 weeks plus prednisone (10 mg/d)	113:115	70.7:70.8	8:8	70.9:100	NR

FIRSTANA NCT01308567	RCT	159 centers in 25 countries	51	Cabazitaxel (20 mg/m^2^)/3 weeks plus prednisone (10 mg/d)	Docetaxel (75 mg/m^2^)/3 weeks plus prednisone (10 mg/d)	389:391	NR	NR	NR	0 to 2

WILLIAM BERRY	Phase 3	Multicenter in the United States	21.8	Mitoxantrone (12 mg/m^2^)/3 weeks plus prednisone (10 mg/d)	Prednisone (10 mg/d)	56:63	70:74	NR	56.7:71.0	0 to 2

PSA: prostate specific antigen; ECOG PS: Eastern Cooperative Oncology Group Performance Status; NR: not reported; RCT: randomized controlled trial. ^*∗*^Intervention versus control.

## Supplementary Materials

Supplementary Materials Supplemental Table 1. The protocol adhered strictly to the preferred reporting items for systematic reviews and meta-analyses (PRISMA) statement. Supplemental Figure 1. Quality and bias assessment of the included clinical trials. Quality and bias of publications of included articles were assessed using the Cochrane Risk of Bias tool. Supplemental Figure 2. Serious adverse events among all the clinical trials. RR is used for adverse events. RR lower than 1 favors treatment. RR is the risk ratio. Supplemental Figure 3. Pooled analysis of abiraterone and enzalutamide. HRs are used for overall survival and radiographic progression-free survival, PSA-progression free survival, and health-related quality of life. HRs lower than 1 favor treatment group. PSA is the prostate-specific antigen and HRs are the hazard ratios.
